# Analyzing the dynamics of complicated and uncomplicated appendicitis during the COVID-19 pandemic in Seoul, Korea: a multifaceted time series approach

**DOI:** 10.4178/epih.e2024081

**Published:** 2024-10-01

**Authors:** Kiook Baek, Chulyong Park

**Affiliations:** 1 Department of Preventive Medicine, Dongguk University College of Medicine, Gyeongju, Korea; 2 Department of Occupational and Environmental Medicine, Dongguk University Gyeongju Hospital, Gyeongju, Korea; 3 Department of Medicine, Graduate School of Kyungpook National University, Daegu, Korea; 4 Department of Occupational and Environmental Medicine, Yeungnam University Hospital, Daegu, Korea; 5 Department of Preventive Medicine and Public Health, Yeungnam University College of Medicine, Daegu, Korea

**Keywords:** COVID-19, Appendicitis, Appendectomy, Seoul

## Abstract

**OBJECTIVES:**

This study investigated the impact of the coronavirus disease 2019 (COVID-19) pandemic and associated control strategies on the incidence of appendicitis in Seoul, using data from 2018 to 2020 from Korea’s National Health Insurance.

**METHODS:**

We analyzed records of total, complicated, and uncomplicated appendicitis cases, as well as the ratio of complicated to uncomplicated appendicitis, using natural spline and piecewise regression models to identify trends and breakpoints. Bayesian structural time-series (BSTS) models were used to evaluate the causal impact of social distancing on appendicitis incidences.

**RESULTS:**

The spline regression analysis indicated decreasing trends in both total and uncomplicated appendicitis cases. Conversely, the incidence of complicated appendicitis and the ratio of complicated to uncomplicated cases increased. Breakpoints for a decline in uncomplicated appendicitis and a rise in the ratio occurred at 31 weeks in 2020 (95% confidence interval [CI], 23.2 to 38.8) and at 33.9 weeks (95% CI, 28.3 to 39.6), respectively. The BSTS model demonstrated a 7.8% reduction in total appendicitis cases (95% credible interval [CrI], -12.1 to -3.3). It also showed a 17.4% decrease in uncomplicated cases (95% CrI, -22.2 to -12.3) and increases of 12.8% (95% CrI, 4.9 to 22.0) in complicated cases and 39.0% (95% CrI, 27.0 to 53.3) in the ratio of complicated to uncomplicated appendicitis.

**CONCLUSIONS:**

The COVID-19 pandemic resulted in a decrease in both total and uncomplicated appendicitis cases, while the number of complicated cases increased. Reduced medical visits likely accounted for these changes. Strategies are needed to manage changes in disease pathophysiology resulting from altered healthcare utilization during health crises.

## GRAPHICAL ABSTRACT

[Fig f5-epih-46-e2024081]

## Key Message

Several studies have reported changes in the epidemiology of acute appendicitis during the COVID-19 pandemic; this study aimed to confirm these findings using national health insurance claims data. Time-series analysis of the epidemiological shift points showed that the decrease in uncomplicated appendicitis cases and the shift in the proportion of complicated appendicitis cases occurred around the time social distancing measures were implemented. When comparing the epidemiology of acute appendicitis before and after the onset of social distancing, there was a significant decrease in the incidence of total acute appendicitis and uncomplicated cases, with a significant increase in complicated appendicitis cases.

## INTRODUCTION

The coronavirus disease 2019 (COVID-19) pandemic has posed unprecedented challenges to public health and healthcare systems globally. The swift proliferation of the disease has impacted healthcare access and the management of other health conditions, with a significant emphasis on controlling infectious diseases [1]. Notably, the increased burden on healthcare facilities and changes in patients’ patterns of visiting medical facilities have affected the diagnosis and treatment of various non-communicable diseases, particularly those requiring surgical interventions [2-4].

It is well-documented that the COVID-19 pandemic has significantly impacted healthcare accessibility and utilization, leading to changes in the severity and outcomes of various conditions that require surgical intervention. Notably, several surgical diseases that are typically manageable with early intervention have seen an increase in severe cases due to delays in treatment [5-7]. These trends have been reported for various surgical conditions, highlighting cases that could have been prevented under normal circumstances in the healthcare system without the pressures of COVID-19. This phenomenon warrants significant public health attention. Acute appendicitis, a quintessential example of a surgical abdomen that typically requires prompt surgical treatment, has also exhibited this pattern [8,9]. Early intervention in acute appendicitis is generally straightforward, but delays can lead to complex surgical procedures or even death, and such cases of delayed treatment reflect the broader impact of the pandemic on surgical outcomes. Some studies have reported a decrease in the incidence of uncomplicated appendicitis, hypothesizing that this trend may be due to delayed surgery and the natural resolution of mild appendicitis cases [10]. Despite the severe strains on healthcare systems during the pandemic, some reports suggest that the clinical presentation of appendicitis has remained consistent and that the rate of hospital-acquired infections has not significantly increased with proper management [11,12]. However, other studies have reported a decrease in appendicitis diagnoses and an increase in complications during the same period [13-15]. This discrepancy may stem from differences in healthcare systems and COVID-19 policies across different regions. Therefore, examining appendicitis outcomes in a densely populated area like Seoul, where healthcare access is highly efficient, COVID-19 infection rates are high, and social distancing policies are stringent, could provide valuable insights. However, there is a notable gap in research at the population level, beyond just a single city or individual medical institution, regarding how the frequency and severity of appendicitis have been influenced by COVID-19 in Korea—a country that adopted a strict approach to COVID-19 response [16] potentially affecting medical service usage patterns [17]. Additionally, differences in healthcare systems across countries could influence these phenomena, especially concerning accessibility to surgical treatments for such diseases.

The aim of this study was to analyze the number of appendicitis surgeries performed in Seoul before and after the COVID-19 pandemic, utilizing data from Korea’s National Health Insurance Service (NHIS). We investigated changes in the proportion of complicated appendicitis cases. This research will significantly contribute to understanding the impact of COVID-19 on the incidence and progression of non-infectious diseases in Korea. Furthermore, this study presents a crucial case for evaluating the effects of COVID-19 on medical accessibility and patient healthcare behaviors.

## MATERIALS AND METHODS

### Definition and collection of data

In Korea, enrollment in the NHIS is mandatory to ensure coverage for medical treatment. The NHIS, a government entity, maintains comprehensive medical records within its system. We accessed secondary data from the NHIS, excluding any personally identifiable information. The dataset spanned the years 2018 to 2020, focusing on cases that met our operational definition of appendicitis. We analyzed the records of patients in Seoul who underwent appendectomies between January 2018 and December 2020.

The operational definition for categorizing cases into total acute appendicitis and complicated appendicitis was based on the Korean Classification of Diseases, which aligns with the International Classification of Diseases, 10th revision (ICD-10), used for insurance claims. A case was included in the study if it involved a surgical procedure following the assignment of a specific diagnostic code for appendicitis. Cases where surgery was performed without a corresponding diagnostic code, or where only a diagnostic code was present without subsequent surgery, were excluded.

To identify all cases of acute appendicitis, we used the following codes: K35 for acute appendicitis, K36 for other appendicitis, and K37 for unspecified appendicitis. For cases of complicated appendicitis, we selected code K35.0 for acute appendicitis with localized peritonitis identified in 2010, and code K35.3 for similar cases in subsequent years. The procedural codes for appendectomies were Q2580 for incision of a periappendiceal abscess, Q2861 for appendectomy in cases of simple appendicitis, Q2862 for appendectomy in cases of perforated appendicitis, and Q2863 for removal of an appendiceal abscess. However, although we were provided with subjects assigned specific procedural codes, we did not receive the actual codes themselves. Therefore, we were unable to use these codes to differentiate between complicated and uncomplicated appendicitis. Additionally, cases classified as other or unspecified appendicitis were considered under the assumption that surgical intervention indicated an acute exacerbation.

The data were meticulously organized on a weekly basis, with the number of cases determined by the admission date. We defined the first week of 2018 as January 1-7, the first week of 2019 as December 31, 2018, to January 6, 2019, and the first week of 2020 as December 30, 2019, to January 5, 2020. Our analysis utilized 52 weeks of data for each year.

### Statistical analysis

To investigate the epidemiology of COVID-19, we charted the weekly number of confirmed cases in Seoul, highlighting key milestones including the identification of the first patient and the implementation of social distancing policies.

We initially presented the annual demographics of Seoul, including the counts and proportions of total appendicitis cases and those classified as complicated. Additionally, we provided daily incidence numbers and daily ratios of complicated to uncomplicated appendicitis. Given the dynamic changes in the population over the periods studied, a separate p-value was not calculated.

Appendicitis is known to exhibit seasonality. To visualize trends and fluctuations over time, we applied a natural spline model with four degrees of freedom per year to regress the weekly incidence of appendicitis and graphed the outcomes. For the time-series regression analysis, we utilized different link functions, employing quasi-Poisson regression for count data (weekly total, complicated and uncomplicated appendicitis) and the gamma family log link function for ratios constrained by positive values.

Next, to identify potential shifts in appendicitis trends, we employed linear piecewise regression using the “segmented function” approach to identify a breakpoint. This analysis did not rely on predefined information about specific intervention points. Instead, our goal was to detect any significant changes in the trend and assess whether these changes coincided with the onset of COVID-19 and the implementation of stricter quarantine measures. Our methodology involved fitting two linear regression models to represent temporal changes along the x-axis, with one unknown breakpoint [18]. The “segmented” function facilitated the modeling of piecewise regression across the entire interval. We utilized the R package “segmented” for this purpose. The algorithm automatically determined the initial breakpoint, with the number of breakpoints limited to one. We set the maximum number of iterations at 10, the tolerance for convergence at 0.00001, and the bandwidth for local linear fitting at 0.1.

We then considered this breakpoint, which was deemed related to the spread of COVID-19, as an intervention point to assess the causal effect using a Bayesian structural time-series model (BSTS). The BSTS method generates counterfactual outcomes using data from a period prior to a specific intervention, allowing for comparison with the actual outcomes following the intervention. Compared to the traditional interrupted time series with segmented regression, BSTS is simpler to implement, requires fewer assumptions, and offers more flexibility in model formation, making it increasingly popular [19]. By accounting for local trends and seasonality, the predicted counterfactual outcomes enable analyses similar to the difference-in-differences approach. This method is widely applied in fields such as economics and public health [20]. In this study, the model produced a counterfactual scenario in a synthetic control series to estimate the intervention’s impact. The analysis utilized time series data, with a pre-intervention period from the 1st week of 2018 to the 33rd week of 2020, and a post-intervention period from the 34th week of 2020 to the 52nd week of 2020. We assume that the outcome time series can be explained by a set of control time series that were not influenced by the intervened period. Additionally, it is assumed that the relationship between the treated series and the control series remains stable throughout the post-intervention period [21]. The R package “CausalImpact” was employed for the BSTS model in the study. The package sets the unobserved state based on the initial observation of the target series and the variance of the dataset. This package employs spike and slab priors for the regression component of the models and utilizes the Kalman filter for the time series component [22]. The performance of each model was presented using the standard deviation (SD) of the residuals (the posterior mean of the residual SD parameter), the SD of the predictions (the SD of the one-step-ahead prediction errors for the training data), and the R² value [23].

Based on data from the first week of 2018, we calculated the excess of actual occurrences compared to the forecasted occurrences post-intervention and determined whether this cumulative value was statistically significant. This was also represented graphically. We accounted for seasonality by considering 52 weeks per year and performed 5,000 iterations.

As part of a sensitivity analysis, we performed a BSTS analysis using data from the fourth week of 2020, which marked the introduction of COVID-19 into Korea, as the interruption point. Additionally, we stratified the analysis of all appendicitis cases by age, separating individuals over and under 20 years old.

The analysis was conducted using version 4.2.0 of the R project, available at https://R-project.org. The “splines” package facilitated the use of spline link functions.

### Ethics statement

The study protocol for the present research was reviewed and approved by the Institutional Review Board of Yeungnam University (No. YUMC 2024-01-023).

## RESULTS

**Figure 1.**
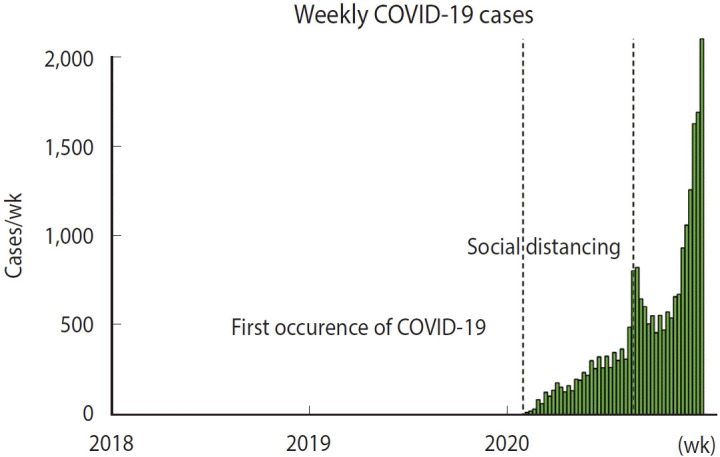
Time series graph of weekly coronavirus disease 2019 (COVID-19) incidence rates in Seoul. The graph includes dashed lines indicating the onset of the first COVID-19 case and the implementation date of the social distancing policy due to the surge in COVID-19 cases.

The first recorded case of COVID-19 in Korea was documented in the fourth week of 2020. A significant increase in cases followed, leading to the initiation of social distancing measures in the 32nd week (Figure 1).

In 2018, there were 13,038 cases of acute appendicitis, representing 0.13% of the population for that year; in 2019, the number slightly increased to 13,352 cases (0.13%), and then decreased to 12,148 cases (0.12%) in 2020 (Table 1). The incidence of complicated appendicitis in 2018 was 3,877, making up 29.7% of all appendicitis cases. This percentage rose to 31.9% in 2019 with 4,264 cases, and further to 36.0% in 2020 with 4,378 cases. The average number of appendicitis cases per week was 250.70 in 2018, 256.70 in 2019, and 233.60 in 2020. For complicated appendicitis, the weekly averages were 74.60 in 2018, 82.00 in 2019, and 84.29 in 2020 (Table 1).

**Figure 2.**
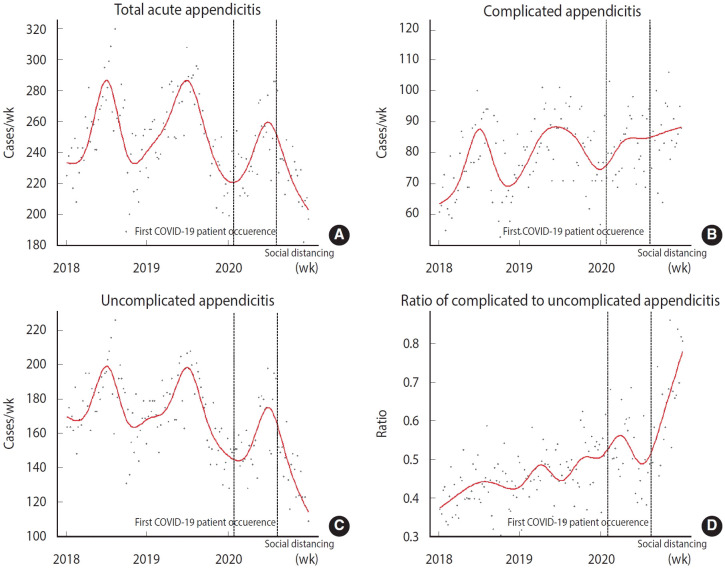
Graph showing the weekly counts of total (A), complicated (B), and uncomplicated (C) appendicitis cases, along with the ratio of complicated to uncomplicated cases (D). Weekly counts are marked with dots, and these data points are fitted with a natural spline curve, represented by a red line. COVID-19, coronavirus disease 2019.

Visualization of appendicitis incidence using natural splines highlighted a clear seasonal pattern, although this pattern was less pronounced for ratios. Complicated appendicitis exhibited a seasonal decline towards the end of 2018 and 2019, followed by an overall rise in 2020. Notably, the ratios surged sharply after mid2020 (Figure 2).

**Figure 3.**
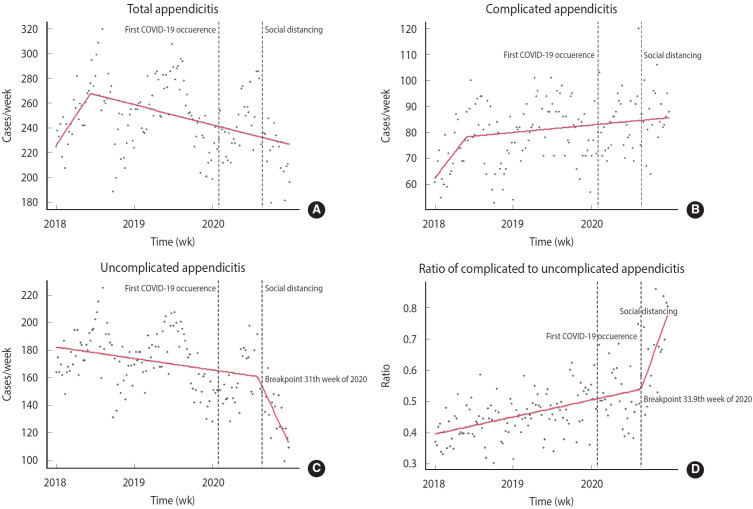
Piecewise regression graph identifying change points in the weekly counts of total (A), complicated (B), uncomplicated (C) appendicitis, and the ratio of complicated to uncomplicated appendicitis (D). Statistical change points occurring after the introduction of coronavirus disease 2019 (COVID-19) are indicated on the graph.

Piecewise regression identified a breakpoint for uncomplicated appendicitis at week 31.0 of 2020, with a 95% CI of 23.2 to 38.8. A trend increase breakpoint for the ratio of complicated to uncomplicated appendicitis was determined at 33.9 weeks of 2020 (95% CI, 28.3 to 39.6), coinciding with the implementation of the government’s social distancing policies in response to a rapid rise in COVID-19 cases. No post-COVID-19 breakpoints were identified for either complicated or total appendicitis cases (Figure 3). The breakpoint for total appendicitis was observed in the 24th week of 2018 (95% CI, 13.5 to 34.49), and for complicated appendicitis in the 22nd week of 2018 (95% CI, 7.4 to 36.6).

**Figure 4.**
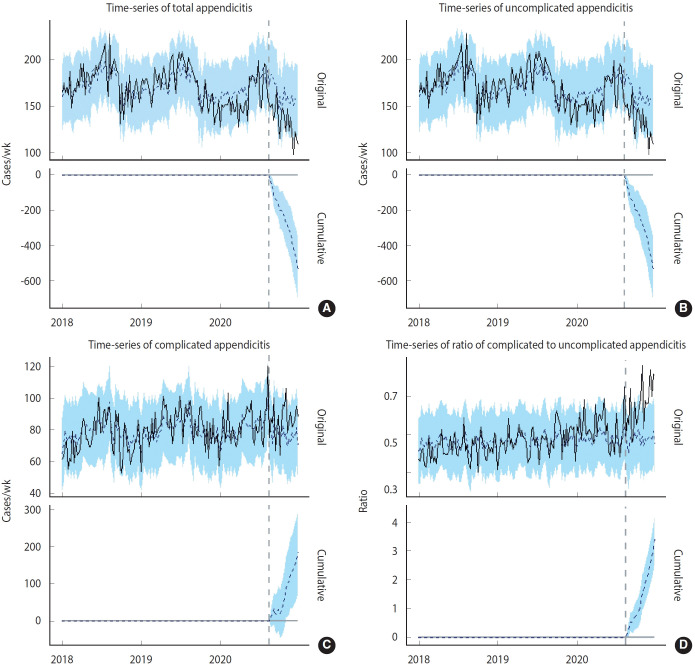
Time series graph using Bayesian structural time series to demonstrate the causal impact of social distancing policies introduced in response to the spread of coronavirus disease 2019 on appendicitis-related metrics. This graph includes series for total (A), complicated (B), and uncomplicated appendicitis (C), as well as the ratio of complicated to uncomplicated cases (D), highlighting significant changes after policy implementation.

Considering the similarity of the breakpoints, BSTS modeling focused on the 34th week of 2020. The incidence of total appendicitis decreased significantly by 7.8 (95% credible interval [CrI], -12.1 to -3.3; posterior tail-area probability= 0.0002). The SD of residuals was 0.75, the SD of predictions was 0.92, and the R² value was 0.43. The incidence of uncomplicated appendicitis decreased by -17.4 (95% CrI, -22.1 to -12.3; posterior tail-area probability= 0.0002). The SD of residuals was 0.78, the SD of predictions was 0.90, and the R² value was 0.39. Conversely, the incidence of complicated appendicitis increased by 12.8 (95% CrI, 4.9 to 22.0; posterior tail-area probability=0.0010). The SD of residuals was 0.88, the SD of predictions was 0.22, and the R² value was 0.22. The ratio of uncomplicated to complicated appendicitis increased by 39.0% (95% CrI, 27.0 to 53.3; posterior tail-area probability = 0.0002) (Table 2 and Figure 4). The SD of residuals was 0.95, the SD of predictions was 0.90, and the R² value was 0.09. Similar outcomes were confirmed using the fourth week of 2020 as the base point, as detailed in the Supplementary Material 1. We stratified the analysis of total acute appendicitis epidemiology by age groups, under and over 20 years, and observed consistent results across both groups (Supplementary Material 2).

## DISCUSSION

In this study, we employed spline regression, piecewise regression, and BSTS models to explore the relationship between the emergence and spread of COVID-19 in Seoul and the incidence of appendicitis, yielding significant findings. The results reveal that while the incidence of uncomplicated appendicitis has decreased, the incidence of complicated appendicitis has increased. As a result, the total number of cases has declined, primarily due to the decrease in uncomplicated appendicitis. However, the ratio of complicated to uncomplicated cases has increased.

Two primary conclusions were drawn from this study. First, the rapid spread of COVID-19 and the implementation of social distancing guidelines by the government coincided with epidemiological changes in appendicitis case numbers. Second, during the COVID-19 pandemic, the incidence rates of total acute appendicitis and uncomplicated appendicitis significantly decreased in Seoul, while the incidence of complicated appendicitis significantly increased. According to the social distancing guidelines, which escalated to level 2 on August 16, 2020 (the 33rd week of the year) in Seoul, stricter measures were enforced. These included restrictions on gatherings at restaurants and cafes after 9 p.m. During this period, policies were enacted to strictly regulate social activities and discourage outdoor activities and gatherings. For example, indoor gatherings of more than 50 people and outdoor gatherings of more than 100 people were banned. Professional sports events could not host spectators. Remote work arrangements were also recommended in workplaces [16]. These social controls, coupled with the fear of infectious diseases, contributed to a reluctance to visit hospitals, leading to unmet healthcare needs among those requiring medical attention [24,25]. Furthermore, there have been ongoing global concerns that the overload of healthcare facilities and personnel due to COVID-19 could reduce the availability of necessary treatments for other critical conditions [26]. Similarly, during the COVID-19 pandemic in Korea, the rate of transfers from emergency rooms to intensive care units and the in-hospital mortality rate increased, underscoring the same issue [27].

The epidemiological changes in appendicitis are likely influenced by shifts in healthcare utilization patterns and alterations in the capacity of medical facilities. The increased burden on the healthcare system, coupled with patients’ avoidance of hospital visits due to the COVID-19 pandemic, may have delayed the timely diagnosis and treatment of appendicitis. This delay could increase the risk of simple appendicitis progressing to complicated appendicitis. Indeed, systematic reviews and meta-analyses have reported an increase in the incidence of complicated appendicitis, associated with delays in surgery during the pandemic [8,28]. Similar outcomes have been reported in several single-institution studies conducted in Korea. One study comparing patients from November 2019 to November 2020 with those from November 2018 to October 2019 reported longer surgery times, more severe inflammation, and an increase in the rate of emergency room visitation, with a difference in time to surgery between 9.0 hours and 17.3 hours [29]. Another institution found that the time from admission to surgery in the emergency room was 186.16 minutes longer [30], and other studies reported differences in the time from symptom onset to hospital arrival [31,32]. However, this hypothesis might explain the increase in the incidence of complicated appendicitis but not the overall decrease in the incidence of appendicitis.

Although acute appendicitis is easily treatable in its early stages, it can lead to serious complications such as perforation and abscess formation if not addressed promptly. These complications can complicate surgical procedures, prolong hospital stays, and potentially lead to mortality if not managed effectively. Numerous studies have documented significant shifts in the epidemiology and prognosis of acute diseases during the COVID-19 pandemic, especially when delays in receiving appropriate treatment or surgical intervention led to acute exacerbations. For instance, in cases of acute cholecystitis, there was an increase in non-surgical treatments, which resulted in longer hospital stays and elevated mortality rates [5]. Similarly, the pandemic saw a significant rise in the number of severe cases of intracranial aneurysm ruptures, accompanied by longer hospital stays [6]. In pediatric intussusception, although the incidence decreased, the severity of cases increased, mirroring trends observed in our study [7]. Indeed, there has been a reported increase in non-accidental mortality during the COVID19 era [33], and the worsening prognosis of various acute diseases, marked by an increase in severity, likely contributed to this trend.

Ironically, the decrease in the incidence of appendicitis, particularly uncomplicated cases, may have resulted from the combined effects of healthcare system pressures and patients’ reluctance to visit hospitals during the COVID-19 era. The term “pressure on the healthcare system” refers to the overwhelming demand for medical resources and personnel needed to manage the surge in COVID-19 cases. Hospitals and clinics were stretched to their limits, with many reallocating staff and equipment to handle the influx of COVID-19 patients. This shift in focus often resulted in a reduced capacity for treating non-COVID-related conditions, such as mild cases of appendicitis. Consequently, reduced healthcare accessibility may have allowed some cases of mild appendicitis to resolve naturally, especially in patients with mild symptoms. Several studies have suggested that spontaneous resolution of acute appendicitis is not uncommon [34]. A previously conducted systematic review and meta-analysis revealed a significant decrease in the incidence of uncomplicated appendicitis but not a significant increase in that of complicated appendicitis, suggesting that delayed treatment of mild, uncomplicated appendicitis could lead to resolution [10]. Nonetheless, our study showed a significant but small increase in the incidence of complicated appendicitis, compared with the decrease in the incidence of uncomplicated appendicitis, indicating that this hypothesis does not fully explain our findings.

Limitations on the use of medical resources and patients’ reluctance to visit hospitals during the pandemic may have led to the performance of only essential surgeries, potentially reducing instances of overdiagnosis, which were common before the pandemic. In this study, the operational definition of appendicitis did not rely on a pathological diagnosis; instead, cases were defined as appendicitis if surgery was performed. Consequently, cases of negative appendectomy might have been included as instances of appendicitis. A multicenter study in Korea reported a negative appendectomy rate of 4.1% [35], where patients suspected of having acute appendicitis underwent surgery, but histopathological examination did not confirm the diagnosis. Similar rates have been reported in the United States [36]. False positives are possible in the diagnosis of any disease, including appendicitis [37-39]. This interpretation, coupled with the potential for natural resolution of mild appendicitis, has been proposed to explain the decrease in the proportion of uncomplicated cases [40]. Although the reduction in overdiagnosis rates cannot fully explain the observed changes in the incidences of total and uncomplicated appendicitis, it may have partially influenced the results. Ultimately, the increase in the rate of natural resolution and the decrease in overdiagnosis rates may stem from the complex interplay between limited medical resources and reduced healthcare accessibility.

Additionally, the spread of COVID-19 and the associated reduction in outdoor activities may have contributed to a lower prevalence of various infectious diseases, potentially including a decrease in gastrointestinal infections. Several studies have noted a reduction in GI infections during the pandemic [41,42]. Gastrointestinal infections, which are considered part of the pathophysiology of appendicitis [36,43,44], may have decreased due to changes in hygiene practices and reduced outdoor activities, leading to a lower incidence of appendicitis. This hypothesis could be explored further in a comprehensive study examining the effects of lifestyle changes on health during the pandemic. We propose that the observed increase in the incidence of complicated appendicitis, coupled with a decrease in uncomplicated cases, can be attributed to a combination of these factors.

During a pandemic such as COVID-19, it is crucial to provide accurate information and guidelines to the public while ensuring that healthcare services remain uninterrupted. The pandemic itself influences healthcare utilization; however, misinformation and fear of infection can further deter people from using medical facilities. This study confirmed that healthcare utilization and the incidence of complications related to acute appendicitis—a condition that requires prompt surgical intervention—varied among Seoul citizens during different periods of social distancing throughout the pandemic. Consequently, it is necessary to allocate healthcare facilities and resources appropriately during a pandemic or when an outbreak occurs. Swift and appropriate actions by health authorities, coupled with public education on correct medical information and utilization, are essential. To tackle these challenges, integrated response plans should be developed. These plans must involve coordination among various levels of government, healthcare providers, and community organizations to ensure a cohesive and effective response. Further research is required to develop suitable response strategies for pandemic situations.

This study has several limitations. First, it relies on secondary data without verifying the definitions of diseases. Despite this, the specificity is considered high due to the utilization of actual surgical case data. Second, the research was confined to Seoul, which complicates the generalization of findings to other regions due to Seoul’s distinct population density and urban characteristics. Third, our data collection was limited to counts, which hindered our ability to control for other variables or conduct detailed subgroup analyses. While we did obtain age-specific total appendicitis data, the NHIS’s policy on data extraction limited our use of count data for other variable-based subgroups and age-specific analyses of perforation presence when daily counts were low enough to potentially identify individuals, thereby preventing a comprehensive subgroup analysis.

Despite these limitations, this study significantly contributes to our understanding of the epidemiological changes in the incidence of appendicitis in Seoul, Korea, during the COVID-19 era. The findings reveal a decrease in the number of relatively mild cases of uncomplicated appendicitis and an increase in the number of complicated cases. These insights are crucial for public health policy decisions and the management of healthcare systems in the context of infectious disease outbreaks and stringent containment measures. Future research should include broader studies that incorporate data from other regions and countries and should investigate the risk factors and pathophysiology of appendicitis more deeply. Additionally, further studies on the impact of the COVID-19 pandemic on other non-infectious diseases are necessary. Such research could greatly enhance the operation of healthcare systems and patient management strategies, and aid in developing response strategies for similar public health crises in the future.

## Figures and Tables

**Figure f5-epih-46-e2024081:**
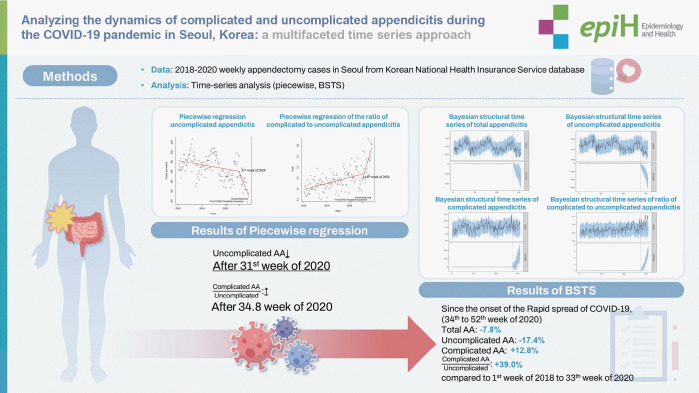


**Table 1.** Descriptive statistics of appendicitis incidence in Seoul, 2018-2020^1^

Year	Population of Seoul	Total acute appendicitis (case/yr)	Complicated appendicitis (case/yr)	Total acute appendicitis (case/wk)	Complicated appendicitis (case/wk)	Ratio (wkly complicated to uncomplicated appendicitis)
2018^2^	10,049,607	13,038 (0.13)	3,877 (29.7)	250.70±26.75 (189, 320)	74.60±11.59 (53, 100)	0.42±0.06 (0.30, 0.86)
2019^3^	10,010,983	13,352 (0.13)	4,264 (31.9)	256.70±24.97 (201, 308)	82.00±10.58 (54, 101)	0.47±0.07 (0.34, 0.63)
2020^4^	9,911,088	12,148 (0.12)	4,378 (36.0)	233.60±23.76 (180, 286)	84.19±10.91 (64, 120)	0.58±0.11 (0.38, 0.86)
Total	-	38,538	12,519	247.00±27.03 (180, 320)	80.30±11.80 (53, 120)	0.49±0.11 (0.30, 0.86)

1 The annual counts are presented as number (%); The annual total acute appendicitis rate is the rate relative to the population; The annual complicated appendicitis rate is calculated with reference to the total number of appendicitis cases; Weekly counts and ratios are presented as mean±standard deviation (minimum, maximum).

2 2018. 01.01 to 2018.12.30.

3 2018.12.31 to 2019.12.29.

4 2019.12.31 to 2020.12.27.

**Table 2.** Changes in acute appendicitis epidemiology following the rapid spread of COVID-19 in Seoul, analyzed by Bayesian structural time series model^1^

Variables	Relative effect [95% CrI] (%)	Absolute average effect/wk [95% CrI]	Absolute cumulative effect (34-52th wk of 2020) [95% CrI]	Posterior tail-area probability	Posterior probability of a causal effect (%)
Total acute appendicitis	-7.8 [-12.1, -3.3]	-18.8 [-30.1, -7.5]	-357.5 [-57.12, -141.7]	0.0002	99.98
Uncomplicated appendicitis	-17.4 [-22.1, -12.3]	-28.2 [-37.8, -18.6]	-536.5 [-718.5, -353.8]	0.0002	99.98
Complicated appendicitis	12.8 [4.9, 22.0]	9.6 [4.0, 16.0]	182.4 [76.9, 295.6]	0.0010	99.90
Complicated appendicitis to uncomplicated appendicitis	39.0 [27.0, 53.3]	0.18 [0.14, 0.23]	-	0.0002	99.98

COVID-19, coronavirus disease 2019; CrI, credible interval.

1 Breakpoint: 34th week of 2020.

## Supplementary materials

Supplementary Material 1. The change of acute appendicitis epidemiology after the point of occurrence of the first COVID-19 patient

Supplementary Material 2. Subgroup analysis of Bayesian structural time series modeling of total acute appendicitis, stratifying by age
