# Supplementary material for: Analyzing the dynamics of complicated and uncomplicated appendicitis during the COVID-19 pandemic in Seoul, Korea: a multifaceted time series approach
Source: Epidemiol Health. 2024 Oct 1;46:e2024081. doi: 10.4178/epih.e2024081 (PMC11832239; doi:10.4178/epih.e2024081)
Supplement: Supplementary Material 1. — The change of acute appendicitis epidemiology after the point of occurrence of the first COVID-19 patient [file epih-46-e2024081-Supplementary-1.docx]

| Supplementary Material 1. The change of acute appendicitis epidemiology after the point of occurrence of the first COVID-19 patient | | | |  |  |
| --- | --- | --- | --- | --- | --- |
|  | Relative effect [95% CI] | Absolute average effect per week [95% CI] | Absolute cumulative effect (4-52th weeks of 2020) [95% CI] | Posterior tail-area probability | Posterior probability of a causal effect |
| Total acute appendicitis | -7.8% [-10.2%, -5.2%] | -20.0 [-26.7, -12.9] | -980.4 [-1309.5, -630.8] | 0.0002 | 99.98% |
| Uncomplicated appendicitis | -14.0% [-16.5%, -11.4%] | -24.5 [-29.6, -19.2] | -1200.1 [-1451.0, -942.4] | 0.0002 | 99.98% |
| Complicated appendicitis | 6.4% [1.3%, 11.9%] | 4.9 [1.1, 9.0] | 243.7 [53.9, 440.1] | 0.0072 | 99.28% |
| Complicated appenditicis to uncomplicated appendicitis | 26.3% [19.8%, 33.4%] | 0.12 [0.10, 0.14] | - | 0.0002 | 99.98% |
|  |  |  |  |  |  |
| CI: Credible interval |  |  |  |  |  |
| Interrupted point: 4th weeks of 2020 | |  |  |  |  |
