# Supplementary material for: Analyzing the dynamics of complicated and uncomplicated appendicitis during the COVID-19 pandemic in Seoul, Korea: a multifaceted time series approach
Source: Epidemiol Health. 2024 Oct 1;46:e2024081. doi: 10.4178/epih.e2024081 (PMC11832239; doi:10.4178/epih.e2024081)
Supplement: Supplementary Material 2. — Subgroup analysis of Bayesian structural time series modeling of total acute appendicitis, stratifying by age [file epih-46-e2024081-Supplementary-2.docx]

| Supplementary Material 2. Subgroup analysis of Bayesian structural time series modeling of total acute appendicitis, stratifying by age | | | | | |
| --- | --- | --- | --- | --- | --- |
|  | Relative effect [95% CI] | Absolute average effect per week [95% CI] | Absolute cumulative effect (34-52th weeks of 2020) [95% CI] | Postarior tail-area probability | Posterior probability of a causal effect |
| Total acute appendicitis of <20 year-old population | -23.1% [-30.8%, -13.9%] | -9.2 [-13.4, -4.9] | -175.1 [-255.4, -92.2] | 0.0002 | 99.98% |
| Total acute appendicitis of ≥20 year-old population | -9.4% [-14.3%, -4.1%] | -18.4 [-29.2, -7.5] | -350.2  [-554.0, -141.7] | 0.0004 | 99.96% |
|  | | |  |  |  |

Interrupted point: 34th weeks of 2020

CI: Credible interval
